# Documentary Analysis of the Scientific Literature on Autism and Technology in Web of Science

**DOI:** 10.3390/brainsci10120985

**Published:** 2020-12-14

**Authors:** Noemí Carmona-Serrano, Jesús López-Belmonte, José-Luis Cuesta-Gómez, Antonio-José Moreno-Guerrero

**Affiliations:** 1 Ceuta Autism Association, University of Granada, 51001 Ceuta, Spain; nhoe@correo.ugr.es; 2 Department of Didactics and School Organization, University of Granada, 51001 Ceuta, Spain; ajmoreno@ugr.es; 3 Department of Education Sciences, University of Burgos, 09001 Burgos, Spain; jlcgomez@ubu.es

**Keywords:** autism, ASD, ICT, technology, bibliometric analysis, scientific mapping, SciMAT, web of science

## Abstract

The objective of the study is to track the progression of the scientific literature on autism and the technology applied to this disorder. A bibliometric methodology has been used, based on a co-word analysis. The Web of Science database was chosen to perform the analysis of the literature. A unit of analysis of 1048 publications was configured. SciMAT software was used mainly for document analysis. The results indicate that the first studies appeared in 1992, but it was not until 2009 that the research volume increased considerably. The area of knowledge where these studies were compiled was rehabilitation, which marks the truly therapeutic nature of this type of study. One of the authors with the most studies, as well as the most relevant research, was Sarkar, N. Manuscripts were usually research articles written in English. It could be concluded that research in this field of study focused mainly on interventions carried out through the use of technological resources, with students or young people who present with ASD. This line of research, although not the only one, was the most relevant and the one that had aroused the most interest among the scientific community.

## 1. Introduction

Autism spectrum disorder (ASD) is defined as a set of neurodevelopmental disorders that encompasses a multifactorial perspective. Science reveals that almost 1.5% of the world’s population suffers from this disorder [1]. ASD mainly reflects alterations in the field of socialization of people [2]. In addition, people with ASD can have communication disorders, as well as repetitive and stereotyped behaviors [3]. Likewise, people with ASD can present deficits in different aspects such as executive functioning, sensory perception, and attention, and can even develop depression [4].

Along these lines, people with this disorder may present signs of aggressiveness, challenging behaviors, and restricted interests [5]. In addition, people with ASD can suffer from anxiety disorders [6]. This can lead to emotional problems [7] at a higher level than seen in people considered to have typical development [8]. All of the above can be increased exponentially if the person with ASD has a low cognitive level [9].

In addition, people with ASD may have difficulties planning daily tasks or actions [10]. They may also exhibit alterations in the structure and use of language [11]. People with ASD may also have writing skills at a lower level than other people [12]. All these singularities that make up ASDs can coexist with other types of pathologies, such as intellectual disability and altered sensory processing [13]. However, people with ASD can process colors with a high level of precision [14]. In this sense, they can also show great musical abilities [15].

Despite the wide range of symptoms, the alteration of the sensory plane is postulated as the main change in people with ASD [16]. At a sensory level, people with ASD can have an altered sense of touch, which is a relevant sense for contact between people [17]. Similarly, people with ASD may have unusual responses to the sounds they perceive around them because they do not process external stimuli in the same way as other people [18]. Another limitation can be seen in motor development. People with ASD can have serious difficulties performing certain tasks of daily life. This can affect both their quality of life and autonomy [19]. Continuing on the sensory plane, the sense of sight can also involve different processing, especially with regard to focusing the gaze on human faces. The reason for this is the hypoactivation of the fusiform area of the face [20]. Therefore, people with ASD may present a set of needs that must be attended to by specialists [21,22,23].

Regarding gender, the scientific literature indicates that women show behaviors and attitudes that are not as restricted and repetitive as men. This is due to sex-linked differences in brain networks. In particular, the difference is seen in networks linked to social and corticosteroid integration [24]. Clinically, the diagnosis of ASD is based on phenotype. Despite this, there are no drugs for its treatment. This leads to therapeutic intervention as the most productive measure for the treatment of previously diagnosed disorders [25].

Early treatment through interventions can lead to substantial improvements in the capacities of people with ASD [26,27]. The practices carried out should be based on observation so that people with ASD can imitate the appropriate behaviors modeled by therapists [28]. All of this will allow for the acquisition of new skills that will have a positive impact on their daily life [29].

Research reveals relevant results from those interventions carried out with technological resources. In this sense, technology-mediated interventions provide people with ASD with a number of benefits [30]. Among them, the improvement of responsibility stands out [31]. Likewise, technology in its different formats (electronic resources, mobile applications, and immersive environments) favors the autonomy of people with ASD. Despite the potential for its use in this population, the design and interface of the technology are currently being analyzed. All this is meant to achieve better adaptation to the needs of people suffering from this disorder [32].

Technological interventions through augmented reality, virtual reality, robotic devices, and mobile applications have shown great advantages. These technologies have obtained improvements in social skills, participation with their immediate environment, communication with other people, and the processes of assimilation and retention of information [33,34,35,36]. However, there are still issues that technology has not been able to address, such as the regulation of emotions [37].

Therefore, technology is positioned as a relevant resource to achieve effective interventions in people with ASD. This will bring about various benefits that will result in a positive integration into society [38,39]. In summary, the use of technology in people with ASD has great potential [40].

### Justification and Objectives

In this work, the relationship between the terms “autism” and “technology” (ASD-TECH) is analyzed through a bibliometric perspective of the literature [41,42]. For this, the Web of Science (WoS) database was chosen as the object of study. This choice is due to it being one of the largest databases in the world.

The novelty of this study lies in its innovative analysis technique. Specifically, a performance analysis and scientific mapping of the documents reported on these constructs were carried out. For a correct and adequate analytical procedure, the protocols established in previous impact studies were followed. This will allow for the reduction of bias in the research [43,44].

Therefore, this work focuses on analyzing the significance and evolution of ASD-TECH in the scientific documents collected from WoS. An initial search was carried out in this database, and no work similar to this one was found. The purpose of this study is to reveal to the scientific community the progress, and upcoming trends [45] on ASD-TECH collected in the WoS literature. This will contribute to the establishment of knowledge about the literature concerning ASD and the technology applied to the treatment of this disorder.

The objectives of this study are to (a) trace the research on ASD-TECH in WoS; (b) determine the scientific evolution on ASD-TECH in WoS; (c) Discover the most relevant topics about ASD-TECH in WoS and (d) locate the most representative authors on ASD-TECH in WoS.

## 2. Materials and Methods

### 2.1. Research Design

The research was based on a bibliometric methodology to achieve the objectives. The potential of this methodology is focused on the quantification and comprehensive evaluation of scientific publications [46,47]. The design of this study will allow for a pertinent survey of the existing literature on bounded contexts [48].

Specifically, the research design focuses on an analysis of co-words [49] and on the study of various indicators related to documentary impact (index h, g, hg, and q2) [50]. The different actions carried out during the research development will allow for the generation of maps with nodes to reveal the performance, the location of the conceptual subdomains, and the thematic development [51] linked to ASD-TECH in the WoS database.

### 2.2. Procedure

Following the procedural guidelines for previous research on this type of study, this work has had various phases [52,53]. The first phase focused on selecting the database (WoS). The second phase focused on the concretion of the concepts to carry out the search (autism, ASD, ICT, and technology). The third phase brought together the preparation of the search equation: (“autism” OR “ASD”) (TITLE) AND (“ICT” OR “technolog*”) (TOPIC). The fourth phase consisted of applying the equation in the main WoS collection (SCI-EXPANDED, SSCI, A and HCI, CPCI-S, CPCI-SSH, BKCI-S, BKCI-SSH, ESCI, CCR-EXPANDED, and IC indices).

**Figure 1:**
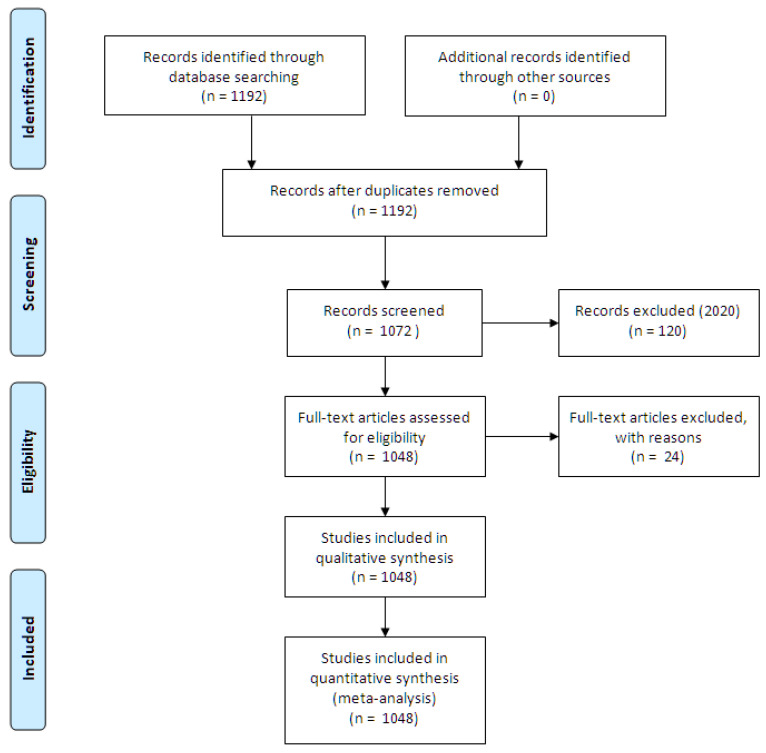
Flowchart according to the PRISMA declaration.

The completion of these phases gave rise to a total of 1192 publications. This documentary volume was then refined using different criteria [54]. The exclusion criteria were: documents published in 2020 (*n* = 120); repeated or poorly indexed documents in WoS (*n* = 24). The application of these criteria produced a final unit of analysis of 1048 publications. Figure 1 contains a flowchart based on the PRISMA protocol that synthesizes the actions carried out.

For the presentation of the results on the performance and scientific production, various inclusion criteria were established [55,56]: Year of publication (all except 2020); Language (x ≥ 10); Publication area (x ≥ 100); Type of documents (x ≥ 100); Organizations (x ≥ 30); Authors (x ≥ 15); Sources of origin (x ≥ 20); Countries (x ≥ 40); the four most cited documents (x ≥ 245).

### 2.3. Data Analysis

Various analytical tools such as Analyze Results, Creation Citation Report, and SciMAT were used. The Analyze Results and Creation Citation Report applications were used to report the year, authorship, country, type of document, institution, language, medium, and most cited documents. SciMAT software was used to carry out the structural and dynamic development of the publications from a longitudinal perspective. The guidelines of previous studies that have used these tools for the correct development of the analysis were followed [57,58].

In addition, SciMAT was used to perform a co-word analysis. This specific analysis is produced in various actions [59]:

- Recognition: It consisted of the analysis of the keywords (*n* = 3829) reported from the extracted publications. In addition, maps of co-occurrence nodes were generated. A normalized network of co-words was made. The keywords with the highest significance were selected (*n* = 3543). Outstanding themes and concepts were delimited through a clustering algorithm.
- Reproduction: Various thematic networks were designed, as well as strategic diagrams. These diagrams are made up of four quadrants. The upper right quadrant contains the relevant and motor topics. The upper left quadrant brings together entrenched and isolated issues. The lower left quadrant welcomes the issues in disappearance or projection. The lower right quadrant reveals the underdeveloped and cross-cutting themes. In the performance of reproduction, the principles of density and centrality intervene. Density establishes the internal strength of the network. Centrality determines the level of connection of a network with others [60].
- Determination: The documentary package was articulated in different time periods, with the purpose of analyzing the evolution of the nodes in time. In this study, three periods were configured (P_1_ = 1992–2012; P_2_ = 2013–2016; P_3_ = 2017–2019). The configuration of these periods started from the criterion of similarity of the documentary volume between each of them. To establish the strength of association between the periods, we started from the number of common keywords. However, for the analysis, a single period was used (P_X_ = 1971–2019).
- Performance: Various production indicators linked to inclusion criteria were established [61] (Table 1).

## 3. Results

### 3.1. Scientific Performance and Production

**Figure 2:**
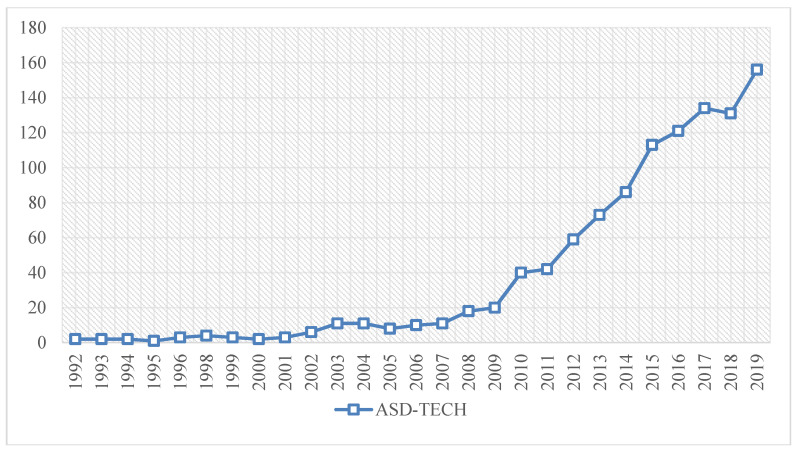
Evolution of research volume.

The first mention of ASD-TECH in WoS dates back to 1992. Since then, studies on this topic have proliferated. This evolution has two clearly differentiated periods. In the first—1992 to 2009—the research volume was relatively low. The second period, 2010 to 2019, was when the research volume grew exponentially year by year. The production peak occurred in 2019 (Figure 2).

Manuscripts dealing with ASD-TECH are mainly written in English. Other languages are used, but these show minimal production with respect to English (Table 2).

Research on ASD-TECH is mostly collected in the area of rehabilitation knowledge, although it is closely followed by the areas of developmental psychology and special education (Table 3).

ASD-TECH studies mostly use research articles to disseminate results. These are followed by conference papers and systematic reviews (Table 4).

There are three institutions that are currently pioneering ASD-TECH research. These are the University of California, Vanderbilt University, and the University of North Carolina (Table 5).

In the study of ASD-TECH, there is one author who has produced, to date, the largest volume of research on the subject. This is Sarkar, N. Two other authors are close behind (Table 6).

The main source of studies on ASD-TECH is the *Journal of Autism and Developmental Disorders*. The research volume is almost double that of *Lecture Notes in Computers Science*, which is the second-largest source (Table 7).

The country with the highest volume of research on ASD-TECH is the United States. Other countries are far behind (Table 8).

Of the four most-cited manuscripts on ASD-TECH (Table 9), the study by Klin et al. (2001) stands out from the rest of the manuscripts with a total of 1137 citations. This research concluded that individuals with autism present abnormal patterns of the visual location at the social level, with less attention to the eyes and more focus on the mouth, bodies, and objects (Table 9).

### 3.2. Structural and Thematic Development

**Figure 3:**
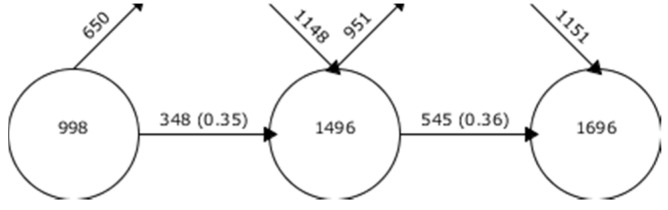
Continuity of keywords between contiguous intervals.

The keywords in each of the collected WoS manuscripts show a disparate trend, over the established periods, in terms of the number of words. In this case, the first period has a much lower volume than the second and third periods. The latter has an even volume of production. Where there is coincidence is in the percentage of coincidence between periods. The percentage is between 35% and 36% (Figure 3). These figures indicate that there is a medium-high volume of coincidence in scientific production. In other words, there are overlapping lines of research in the field of ASD-TECH in the three established time periods.

The analysis of thematic performance, based on various bibliometric indicators, as shown in Table 10, provides information on the topics most researched by the scientific community. In this case, in the first period (1992–2012), the subject matter with the greatest bibliometric value is “children.” In the second period (2013–2016), the topic with the highest bibliometric value is “intervention.” In the last period (2017–2019), the subject with the highest bibliometric value is again “children.” In all these cases, the values of these themes were much higher than the rest.

**Figure 4:**
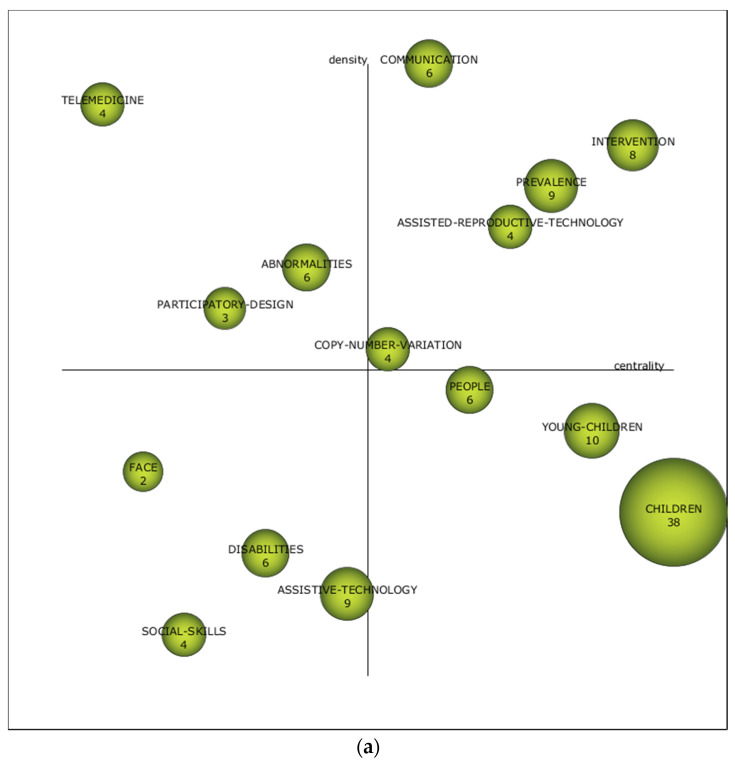

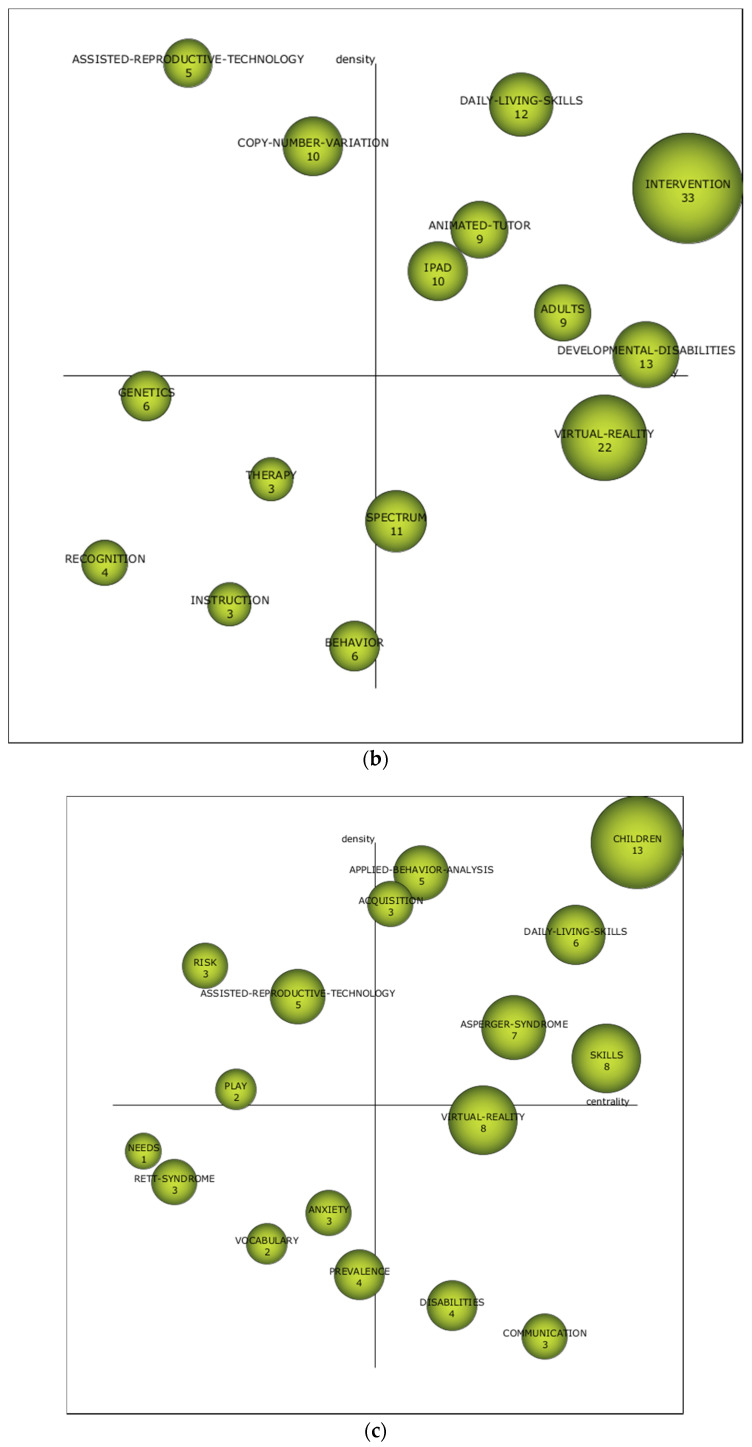
ASD-TECH strategic diagram by h-index. (**a**) 1992–2012; (**b**) 2013–2016; (**c**) 2017–2019.

The strategic diagram, taking into account in this case index h as the main reference, shows the information on the relevance of the different themes in each of the established time periods (Figure 4). Depending on the location of the themes in the diagram, their relevance will be greater or lesser. In this case, the position of the subject is produced, taking into account its external connection (centrality) and its internal connection (density). In addition, the keywords that relate to the driving issues are listed in Appendix A.

In the first period (1992–2012), the themes considered as driving forces were “communication,” “intervention,” “prevalence,” “assisted reproductive technology,” and “copy number variation.” During this period, it can be seen that the main themes were communication with people with ASD, intervention for the improvement of those affected, and the prevalence of representations of ASD in society. It can also be seen that studies focused on assistive technology and chromosome analysis.

In the second period (2013–2016), the driving themes were “daily living skills,” “intervention,” “animated tutor,” “iPad,” “adults,” and “developmental disabilities.” In this period, the relevance of the studies varied slightly with respect to the previous period. In this case, we analyzed the skills needed to be able to function in daily life, interventions to minimize the difficulties caused by ASD, tutors who look after people with ASD, new technological resources such as iPads, adults with ASD, and possible developmental disabilities that can lead to a growth in the number of people with ASD.

In the last period (2017–2019), the motor themes “children,” “applied behavioral analysis,” “acquisition,” “daily living skills,” “Asperger’s syndrome,” and “skills.” In this period, the trends were slightly different again from previous periods. In this case, the most relevant studies focused on children, on the analysis of the behavior of subjects with ASD, on the skills of people with ASD, on the acquisition of competencies for social life, and on Asperger’s syndrome. In addition, since this was the final period, the topics “needs,” “Rett syndrome,” “vocabulary,” “anxiety,” and “prevalence” must also be considered. These themes, due to their position in the diagram, are considered to be unknowns. This is because they may be the future driving force or may be themes that have disappeared from the lines of research established by the scientific community.

### 3.3. Thematic Evolution of Terms

In the evolution of research on ASD-TECH, two aspects can be observed: the main research lines and the connections established between the different topics. The continuous lines show conceptual connections. In other words, these are connections in which the themes themselves contain other themes seen previously. The discontinuous lines show connections by means of keywords. When the width of the line, both continuous and discontinuous lines, is greater, it means there is a greater number of coinciding themes or keywords between themes. Therefore, the wider the line, the closer the relationship.

**Figure 5:**
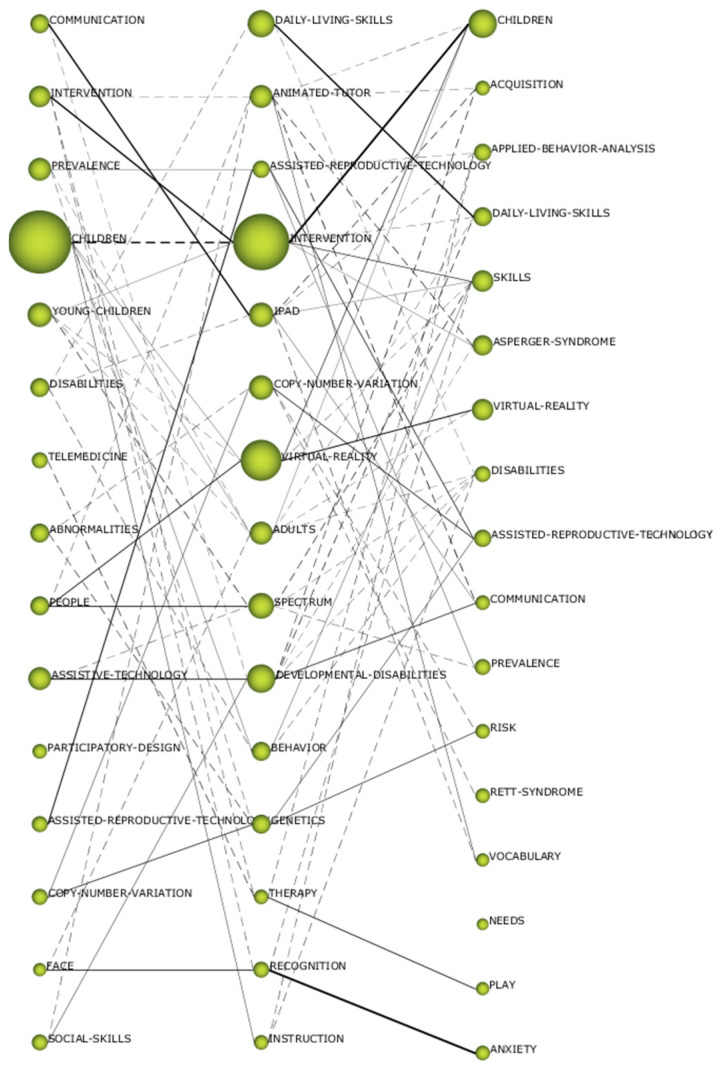
Thematic evolution by h-index.

Analyzing Figure 5, it can be seen that there is a theme that is repeated in all three periods, such as “assistive reproductive technology,” which can be considered a line of research in this field of study. This does not mean that it is the most relevant or the only one. In this case, it can be seen that the mainline of research is “intervention—children.” That is, ASD-TECH research is mainly focused on therapeutic interventions in minors. In this field of study, other lines of research are observed, in addition to those previously established, such as “people–virtual_reality” and “communication—iPad skills,” although they are not as relevant as the one indicated above. One aspect to highlight in the analysis of the figure is that more nonconceptual connections are observed than conceptual ones. This means that the lines of research are differentiated from each other, and so far, there are no implications of the main lines of research for each other.

### 3.4. Authors with the Highest Relevance Index

**Figure 6:**
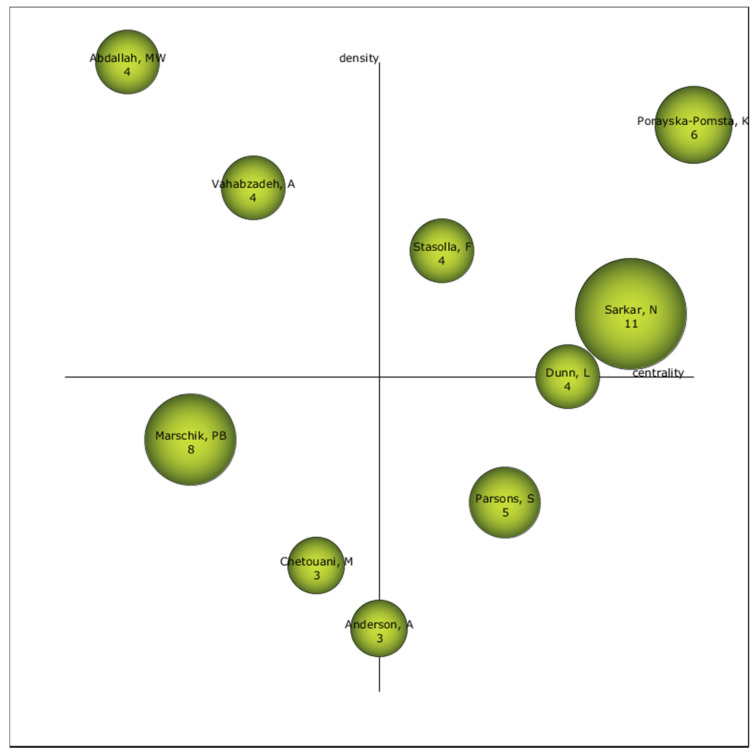
Strategic author diagram of the entire production.

The strategic diagram of scientists in the ASD-TECH branch of the study shows that Porayska-Pomsta, K., Stasolla, F., and Sarkar, N., are the most relevant authors in this line of research. Furthermore, the authors Marschik, P. B., Chetouani, M., and Anderson, A., should be taken into account since their position in the diagram places them as possible relevant authors of ASD-TECH studies in the future (Figure 6).

## 4. Discussion

The purpose of the study was to assess the importance of scientific publications on autism linked to the technological field. We live in a society marked by continuous advances in technology [52]. Innovative and electronic resources are increasingly widespread in all areas of life [44]. For this reason, the need arises to check the state of the links between ASD and technology. This is reflected in the literature, which shows the role of technology in the field of ASD [30,31,32,33,34,35,36,37,38,39,40]. Therefore, given the peculiarities of an increasingly innovative and technological society, it is pertinent to focus on how this disorder is treated from a technological point of view. For this, an analysis was carried out of everything collected in the scientific literature so far.

First, the performance analysis allows for a general evaluation of the studies carried out on the ASD-TECH topic. The first scientific research on ASD-TECH was in 1992. From then until 2019, a differentiated evolution can be observed in two periods: a first period, between 1992 and 2009, when the research was scarce and linear in time. In the second period, from 2010 to 2019, there was a considerable increase in research, with growth year by year. The differentiation of the two periods may be linked to the increase and improvement in technological resources in the social sphere, which was then transferred to other fields, such as the treatment and care of ASD. It should also be noted that the peak of research production was in 2019.

The main type of manuscript used to present scientific results is research articles, which are usually written in English. This shows that the research is grounded in time and that trends in this type of study are related to field studies. In addition, the main area of knowledge, where these studies are collected is rehabilitation. This shows that technological resources are used for the rehabilitation of people with ASD.

There is no single relevant institution that dominates this field of study; the three main centers of research are the University of California, Vanderbilt University, and the University of North Carolina. Among the most noteworthy authors is Sarkar, who, as well as having the greatest volume of research, is one of the most relevant researchers, according to the analyses carried out. In this case, he can be considered a key reference in studies on ASD-TECH. This does not mean he is the only author to be considered; attention should also be paid to Marschik, Chetouani, and Anderson, since they may be among the main researchers in this field of study.

Among the different sources that disseminate to the scientific community the findings on this subject, there is the *Journal of Autism and Developmental Disorders*. Its volume of production is quite high. In addition, the country with the highest volume of production is the United States. Among the most frequently cited articles is that of Klin et al. (2001), which focuses on the attention that people with ASD pay to their social interactions. His citation volume is very high compared to other manuscripts dealing with this line of research.

In terms of the structural and thematic development, it can be seen that the level of coincidence between periods is over 35%. This shows that the scientific community, although it can establish new lines of research, maintains lines of study that are the basis for this type of research.

The thematic performance shows two themes with the highest bibliometric values in this field of study. One is “children,” which is repeated in the first and last periods; the other is “intervention,” which dominated the second period. This indicates how the direction of research on ASD-TECH is focused by the scientific community on children and intervention.

The strategic diagram indicates that there are changes in the driving themes of the three periods analyzed. In the first period, the most relevant studies focus on the communication of subjects presenting with ASD, on the various intervention techniques used with people with ASD, on the prevalence of the population presenting ASD, on assistive technology, and on chromosome studies of people with ASD. In the second period, the most relevant studies focus on the analysis of the skills needed to be able to function in daily life, interventions to minimize the difficulties arising from ASD, the tutors who attend to people with ASD, newly emerging technological resources such as iPads, adults with ASD, and the possible developmental disabilities that can result in a rise in the number of people with ASD. Finally, in the last period, studies focused on children, the analysis of the behavior of people with ASD, the skills of people with ASD, the acquisition of competencies for social life, and Asperger’s syndrome. This indicates that the focus of research has changed. Furthermore, we must take into account the themes of “needs,” “Rett syndrome,” “vocabulary,” “anxiety,” and “prevalence,” which may be the driving forces in this line of research.

Thematic developments have shown that there are more nonconceptual than conceptual connections. This indicates that there are independent lines of research in this field of study. Furthermore, a line of research is shown that remains, at a thematic level, constant over time, as is the case with “assistive reproductive technology,” given that it is the only theme that is repeated in all three periods. Furthermore, the connection established between them is conceptual. Thanks to this theme, this field of study does not present a conceptual gap. However, it turns out that this line of research is not the most relevant one over time. In this case, the main line of research is “intervention—children” because its connections are conceptual and thicker than the other established connections. For this reason, it can be said that research on ASD-TECH focuses mainly on therapeutic intervention in young people who have ASD.

## 5. Conclusions

The main conclusion that can be drawn from this investigation is that the ASD-TECH line of research has become relevant, in terms of production volume, over the last 10 years. Research in this field of study is mainly focused on interventions carried out, through the use of technological resources, with students or young people who present with ASD. This line of research, although not the only one, is the most relevant and the one that arouses the most interest among the scientific community.

This research focuses on offering both researchers and the different groups that attend to people with ASD information on the main trends in ASD-TECH studies.

The main limitations of the study are related to the purity of the database. This requires reading all of the manuscripts in order to know whether they meet the inclusion criteria established in this study. This represents an extra effort for researchers. Another limitation is the purification of the keywords. Many of them are poorly recorded or are presented differently, both by acronyms and by their full names. The authors have reviewed all the keywords, correcting and grouping them as necessary. Finally, another possible limitation of this study is the inclusion criteria, which, if modified, may lead to variations in the data presented. The criteria established in this study were based on the equity and volume of data. To this end, the authors have established various criteria, selecting the most appropriate ones. As a future line of research, field studies can be developed based on interventions through the use of technological resources, such as augmented reality.

## 6. Theoretical and Practical Implications

Both theoretical and practical implications can be drawn from this research. Among the theoretical implications, this study allows us to identify the most relevant and prolific institutions and authors on ASD-TECH, which provides researchers with relevant knowledge to assist with compiling adequate and interesting sources from this line of research. Furthermore, it facilitates the identification of the main scientific journals that publish the results obtained in the ASD-TECH line of research. It should also be noted that knowing the main lines of research generated in this field of study allows scientists themselves to guide the trends. In this case, they do not need to check what has been published previously; thanks to this research, they can know which lines of study they can establish. Finally, it can be said that this study makes it possible to provide more scientific literature on ASD-TECH. Among the practical implications is the fact that most research in this field of study is focused on minors and on interventions. This manuscript can be used by therapists themselves to discover new means of intervention, as well as to identify those interventions with the use of the most relevant technology and with the best results. Furthermore, this study provides families and therapists with the most relevant and significant journals in this field of study, speeding up the search for this type of scholarship.

## Figures and Tables

**Table 1:** Production indicators and inclusion criteria.

Configuration	Values
Analysis unit	Keywords, authors, WoS
Frequency threshold	Keywords: P_1_ = (3), P_2_ = (4), P_3_ = (5)
	Authors: P_X_ = (4)
Network type	Co-occurrence
Co-occurrence union value threshold	Keywords: P_1_ = (2), P_2_ = (2), P_3_ = (2)
	Authors: P_X_ = (2)
Normalization measure	Equivalence index: eij = cij2/root (ci–cj)
Clustering algorithm	Maximum size: 9; minimum size: 3
Evolutionary measure	Jaccard index
Overlapping measure	Inclusion rate

**Table 2:** Scientific languages of publications.

Languages	*n*
English	1039
Spanish	15

**Table 3:** Areas of knowledge.

Areas of Knowledge	*n*
Rehabilitation	216
Developmental psychology	187
Special education	176
Education, educational research	101

**Table 4:** Types of documents.

Type of Document	*n*
Article	677
Proceedings paper	259
Review	104

**Table 5:** Institutions.

Denomination	*n*
University of California	38
Vanderbilt University	37
University of North Carolina	35
Pennsylvania Commonwealth System of Higher Education (PCSHE)	31

**Table 6:** Most prolific authors on the relationship between the terms “autism” and “technology” (ASD-TECH).

Authors	*n*
Sarkar, N.	22
Parsons, S.	20
Warren, Z.	17

**Table 7:** Sources of origin.

Denomination	*n*
*Journal of Autism and Developmental Disorders*	66
*Lecture Notes in Computer Science*	37
*Research in Autism Spectrum Disorders*	26
*Autism*	25
*Journal of Special Education Technology*	25

**Table 8:** Most productive countries in terms of ASD-TECH research.

Countries	*n*
USA	519
England	109
Italy	53
Australia	49
Canada	43

**Table 9:** Most cited articles on ASD-TECH.

References	Citations
Klin, A.; Jones, W.; Schultz, R.; Volkmar, F.; Cohen, D. Visual fixation patterns during viewing of naturalistic social situations as predictors of social competence in individuals with autism. *Archives Gen. Psychiatry* **2001**, *59*, 809–816, doi:10.1001/archpsyc.59.9.809	1137
Miles, J.H. Autism spectrum disorders-A genetics review. *Genet. Med.* **2011**, *13*, 278–294, doi:10.1097/GIM.0b013e3181ff67ba	296
Horner, R.H.; Carr, E.G.; Strain, P.S.; Todd, A.W.; Reed, H.K. Problem behavior interventions for young children with autism: A research synthesis. *J. Autism Dev. Disord.* **2002**, *32*, 423–446, doi:10.1023/A:1020593922901	275
Krumm, N.; O’Roak, B.J.; Shendure, J.; Eichler, E.E. A de novo convergence of autism genetics and molecular neuroscience. *Trends Neurosci.* **2014**, *37*, 65–105, doi:10.1016/j.tins.2013.11.005	248

**Table 10:** Thematic research on ASD-TECH.

**1992–2012**						
**Denomination**	**Works**	**Index h**	**Index g**	**Index hg**	**Index q2**	**Citations**
Children	79	38	71	51.94	59.13	5093
Young children	12	10	12	10.95	23.45	1008
Prevalence	10	9	9	9	19.67	741
Assistive technology	11	9	11	9.95	20.78	516
Intervention	12	8	10	8.94	21.73	489
Disabilities	8	6	8	6.93	9.17	426
Abnormalities	6	6	6	6	24.62	505
Communication	9	6	9	7.35	16.43	323
People	7	6	7	6.48	19.6	330
Assisted reproductive technology	4	4	4	4	11.49	311
Social skills	4	4	4	4	15.36	221
Copy number variation	4	4	4	4	12.65	371
Telemedicine	7	4	6	4.9	14.7	133
Participatory design	4	3	4	3.46	6	38
Face	2	2	2	2	21.91	361
**2013–2016**						
**Denomination**	**Works**	**Index h**	**Index g**	**Index hg**	**Index q2**	**Citations**
Intervention	186	33	45	38.54	36.78	3502
Virtual reality	53	22	34	27.35	29.66	1236
Developmental disabilities	20	13	20	16.12	21.93	560
Daily living skills	19	12	18	14.7	18	365
Spectrum	15	11	15	12.85	14.83	335
iPad	15	10	14	11.83	13.78	260
Copy number variation	15	10	14	11.83	18.44	617
Animated tutor	11	9	10	9.49	19.21	517
Adults	19	9	16	12	15.59	266
Behavior	9	6	8	6.93	8.49	129
Genetics	7	6	7	6.48	8.83	88
Assisted reproductive technology	9	5	8	6.32	10	124
Recognition	5	4	4	4	13.86	170
Therapy	5	3	4	3.46	8.49	57
Instruction	4	3	3	3	8.12	64
**2017–2019**						
**Denomination**	**Works**	**Index h**	**Index g**	**Index hg**	**Index q2**	**Citations**
Children	250	13	19	15.72	15.3	969
Skills	38	8	11	9.38	9.38	158
Virtual reality	21	8	9	8.49	9.38	109
Asperger’s syndrome	17	7	11	8.77	10.91	16
Daily living skills	31	6	9	7.35	8.12	119
Assisted reproductive technology	8	5	8	6.32	8.66	260
Applied behavioral analysis	14	5	8	6.32	6.32	79
Disabilities	15	4	5	4.47	5.29	43
Prevalence	8	4	6	4.9	6.63	48
Communication	16	3	4	3.46	3.87	34
Acquisition	15	3	4	3.46	3.87	28
Risk	6	3	5	3.87	7.35	51
Rett syndrome	4	3	4	3.46	4.24	21
Anxiety	4	3	4	3.46	3.87	23
Vocabulary	3	2	3	2.45	6.32	25
Play	5	2	3	2.45	4.47	19
Need	3	1	1	1	1	1

## Appendix A

**Table A1:** Relationship between keywords and driving themes.

**1992–2012**	
**Driving Themes**	**Keywords**
Communication	Augmentative and alternative communication, feedback, spelling, speech-generating devices, behavioral analysis, language, learning and literacy
Intervention	Vocabulary, multimedia, recognize, adolescents, emotions, skills, Asperger’s syndrome and play
Prevalence	Epidemiology, birth, paternal age, cytokines, disorders, pregnancy, population, and autistic disorder
Assisted reproductive technology	Child and infantile autism
Copy number variation	Structural variation and genetics
**2013–2016**	
**Driving Themes**	**Keywords**
Daily living skills	Video modeling, moderate, video prompting, computer-based intervention, intellectual disability, video, students, and self
Intervention	Autism, young children, individual, adolescents, autism spectrum disorder, skills, technology, and Asperger’s syndrome
Animated tutor	Computer-assisted instruction, emotion recognition, vocabulary, communication skills, computer, virtual environments, meta-analysis, and high-functioning autism
iPad	Picture exchange, literacy, augmentative and alternative communicative, apps, behavioral intervention, parents, speech-generating devices, and communication
Adults	Functioning autism, mind, usability, disorders, youth, attention, social media, and employment
Developmental disabilities	Language, alternative communication, quality of life, assistive technology, mobile technology, social skills, transition, and young adults
**2017–2019**	
**Driving Themes**	**Keywords**
Children	Technology, autism, high-functioning autism, adolescents, adults, autism spectrum disorder, intervention, and spectrum disorders
Applied behavioral analysis	Special education, technology perspectives, academic skills, exceptionality, methodology, outcomes, parents, and telehealth
Acquisition	Alternative communication, preschoolers, augmentative and alternative communication, communication skills, speech-generating devices, tablets, generalization, and language
Daily living skills	Individuals, students, employment, activity schedules, intellectual disability, mobile technology, video, and young adults
Asperger’s syndrome	Emotion recognition, attention deficit hyperactivity disorder, experiences, pervasive developmental disorders, randomized controlled trial, social skills, and computer-assisted instruction
Skills	Social robots, young children, joint attention, behavior, emotions, iPads, serious games, and social interaction
